# On the Physiological Action of Arsenious Acid

**Published:** 1875-06

**Authors:** 


### ARTICLE X.

### *On the Physiological Action of Arsenious Acid.*

Professor Boehm, of Dorpat, records in the *Archiv fur Experimentelle Pathologie and Pharmakologie* vol. ii. Heft 2, his own researches and those of his pupils, on the physiological action of arsenious acid. The experiments were conducted by S. Unterberger upon cats and dogs. On injection of a watery solution of arsenious acid into a vein, a gradual sinking of the mean blood-pressure occurs. The amount of sinking is in direct relation to the quantity of arsenic employed. This sinking is never preceded by an increase of the blood-pressure, and is only temporary when it owes its origin to small doses (0.005 to 0.03 gramme.) At the same time the pulse is rendered slow. These phenomena can be ascribed partly to paralysis of the abdominal blood-vessels, and partly to a diminution of the capacity of the cardiac muscles for action. The cardiac nerves in animals poisoned with arsenic exhibit normal relations. The vessels of the sympathetic areas are not paralyzed by the poison.

The action of this drug upon the intestinal canal was also studied. To arsenious acid is generally ascribed a local irritating action. The chemical reason for this irritating action is quite unknown. This substance exhibits no special affinity either for water or for albuminous bodies. The action of this drug was studied in similar animals (dogs of the same size, age, weight, etc.,) and its effects contrasted according as it was introduced by the mouth or by injection in solution into the circulation. When one has before him two similar animals whch have been poisoned with arsenic, it is impossible from the *post-mortem* appearance to say which of the two animals has received the poison by the stomach or through the blood. Not only so, but the phenomena during life are similar, and the only difference is that the smallest lethal doses when given by the mouth, are not sufficient to kill a similar animal when injected into a vein ; and that in the latter mode of poisoning, death always occurs somewhat later than in poisoning through the stomach.

After death no matter how the poison was introduced, the mucous membrane of the stomach throughout its whole extent was tinged dark red, was considerably swollen, and presented a velvety appearance. The redness was always confined to the most superficial layers of the mucous membrane. In the serous membrane of the stomach, beyond a very pronounced filling of the vessels, numerous large ecchymoses were generally present; loss of the substance of the mucous membrane was never observed. The degeneration of the gastric glands, described by other authors in the rabbit, were not found. Essentially different was the appearance throughout the whole length of the intestinal canal. The mucous membrane was covered throughout its entire extent by a yellow coloured, jelly-like, but still consistent membrane about one millimetre thick. Microscopically, this membrane appeared to consist of innumerable pus-cells, embedded in a structureless material. This membrane could be removed, when the mucous coat was exposed generally filled with small point-like ecchymoses. The villi were greatly swollen, and were devoid of epithelium over their entire surface, and numerous pus-like cells were also embedded in their substance. In the other organs, nothing remarkable was found. The liver and kidneys had never undergone fatty degeneration. Ecchymoses in the endocardium of the left ventricle were constant, often also in the other serous membranes. These results are not favourable to the assumption of a local irritating action of the poison. The authors hold as unexplained the action of sepsin to that of arsenic, in that the former exhibits no local action. In the intestine, only traces of arsenic were found on analysis of its contents by the Marsh method.—*London Med. Record.*

